# Use of Graph Theory to Characterize Human and Arthropod Vector Cell Protein Response to Infection With *Anaplasma phagocytophilum*

**DOI:** 10.3389/fcimb.2018.00265

**Published:** 2018-08-03

**Authors:** Agustín Estrada-Peña, Margarita Villar, Sara Artigas-Jerónimo, Vladimir López, Pilar Alberdi, Alejandro Cabezas-Cruz, José de la Fuente

**Affiliations:** ^1^Facultad de Veterinaria, Universidad de Zaragoza, Zaragoza, Spain; ^2^SaBio, Instituto de Investigación en Recursos Cinegéticos (IREC), CSIC, Universidad de Castilla-La Mancha (UCLM), Junta de Comunidades de Castilla - La Mancha (JCCM), Ciudad Real, Spain; ^3^UMR Biologie Moléculaire et Immunologie Parasitaires (BIPAR), INRA, Agence Nationale de Sécurité Sanitairede l'Alimentation, de l'Environnement et du Travail (ANSES), Ecole Nationale Vétérinaire d'Alfort, Université Paris-Est, Maisons-Alfort, France; ^4^Faculty of Science, University of South Bohemia, Ceské Budějovice, Czechia; ^5^Institute of Parasitology, Biology Center, Czech Academy of Sciences, Ceské Budějovice, Czechia; ^6^Department of Veterinary Pathobiology, Center for Veterinary Health Sciences, Oklahoma State University, Stillwater, OK, United States

**Keywords:** graph theory, network, omics, ras-related proteins, tick, *Anaplasma phagocytophilum*

## Abstract

One of the major challenges in modern biology is the use of large omics datasets for the characterization of complex processes such as cell response to infection. These challenges are even bigger when analyses need to be performed for comparison of different species including model and non-model organisms. To address these challenges, the graph theory was applied to characterize the tick vector and human cell protein response to infection with *Anaplasma phagocytophilum*, the causative agent of human granulocytic anaplasmosis. A network of interacting proteins and cell processes clustered in biological pathways, and ranked with indexes representing the topology of the proteome was prepared. The results demonstrated that networks of functionally interacting proteins represented in both infected and uninfected cells can describe the complete set of host cell processes and metabolic pathways, providing a deeper view of the comparative host cell response to pathogen infection. The results demonstrated that changes in the tick proteome were driven by modifications in protein representation in response to *A. phagocytophilum* infection. Pathogen infection had a higher impact on tick than human proteome. Since most proteins were linked to several cell processes, the changes in protein representation affected simultaneously different biological pathways. The method allowed discerning cell processes that were affected by pathogen infection from those that remained unaffected. The results supported that human neutrophils but not tick cells limit pathogen infection through differential representation of ras-related proteins. This methodological approach could be applied to other host-pathogen models to identify host derived key proteins in response to infection that may be used to develop novel control strategies for arthropod-borne pathogens.

## Introduction

The transcriptional regulatory network coordinates gene expression, resulting in the production of proteins involved in the execution of cellular processes (Xue et al., 2014). Modern biological investigations are based on genome-scale analysis, which implicate thousands of molecular entities as being of interest due to their change in abundance in response to different stimuli. However, the interpretation of these findings represents one of the greatest challenges in modern biology. Gene Ontology enrichment analysis may indicate that a set of genes or proteins are implicated in a given pathway or process, but what role they play or how they function together in response to stimuli is not defined (O'Hara et al., 2016). Protein-protein interaction databases may be used to define proteins that share functional or physical associations, but the information returned generally lacks a biological significance (Barrat et al., 2004). The biological significance has been simulated using for example Petri nets and the signaling Petri nets algorithm to dynamically model flow in simulated protein-protein interactions (O'Hara et al., 2016). However, this approach may emulate the behavior of a system, but the biological context is lost.

In graph theory, a network is a collection of nodes and edges connecting them to represent their relationships (Barrat et al., 2004; Horvath, 2011; Robinson et al., 2015). Other than nodes, relationships linking start and end nodes can also have properties, particularly the interaction strength or edge weight (Barrat et al., 2004; Horvath, 2011; Robinson et al., 2015). Several indices measure network properties from which the relationships are derived. The degree centrality (DC) is the most basic measure of a network, representing the number of edges leaving (or arriving at) a given node after weighting by the total number of records containing this interaction. The DC provides an estimation of the strength of the association but does not evaluate the importance of each node in the context of the network. The Weighted Degree (WD) is the calculation of the relative DC of each node in the context of the complete network. Another centrality index, the node Betweenness Centrality (BNC) indicates how often a node is found on the shortest path between two nodes in the network (Estrada-Peña et al., 2015). The implicit meaning of the BNC is the importance of the node in the flow of other components of the network. Networks have also a measure of Modularity, which explains the nodes that interact more among them than with the other nodes in other clusters. This modularity is obtained from the number of interactions among nodes and the weight of the edges linking them.

Herein, we developed a method to obtain biologically significant information from large datasets of annotated protein representation, specifically focused on how these proteins function together in response to stimuli. The method was based on the development of a metaphorical network that links the representation of dozens of thousands of proteins with various cell processes, from which several indexes describing the graph were obtained. The concepts of graph theory have been commonly used to describe ecological relationships (Jordano et al., 2006) or the behavior of social networks (Girvan and Newman, 2002; Kourtellis et al., 2013), but recent applications in Genomics and Systems Biology have been proposed (Horvath, 2011). Definitions of graph theory terms and its practical use for our method are described in Supplementary Table 1.

Our study was based on the protein profile of uninfected and infected cultured cells of *Ixodes scapularis* tick vector hemocytes (ISE6) and human host neutrophils (HL60), involved in the life cycle of the tick-borne pathogen *Anaplasma phagocytophilum* (Severo et al., 2015; Villar et al., 2015). *A. phagocytophilum* is an obligate intracellular bacterium that causes human granulocytic anaplasmosis, a disease characterized by fever, headache, muscle pains, and pancytopenia (Severo et al., 2015). Recent studies have shown that several biological pathways are modulated during *A. phagocytophilum* interaction of tick vector and human host cells (de la Fuente et al., 2016, 2017; Gulia-Nuss et al., 2016). These pathways include remodeling of the cytoskeleton (Ayllón et al., 2013), gene transcription (Sultana et al., 2010; Ayllón et al., 2015), bacterial intracellular development (Huang et al., 2010a,b), metabolism (Villar et al., 2015; Dumler et al., 2016; Cabezas-Cruz et al., 2017; Taank et al., 2017), stress response (Neelakanta et al., 2010), apoptosis and immune response (Borjesson et al., 2005; de la Fuente et al., 2005; Severo et al., 2013; Ayllón et al., 2015; Shaw et al., 2017), cell cycle (Khanal et al., 2017), and epigenetics (Cabezas-Cruz et al., 2016; Dumler et al., 2018) among others.

The application of this method to tick cell response to infection demonstrated that the resulting network of protein and cell processes has the general properties of a natural network, which supports the validity of the approach. Furthermore, the indexes of Centrality of the network could be used to define the main changes of different tick cellular processes affected in response to infection. In human cells, the Weighted Degree (WD), an index derived from the connections of the network and protein representation was used to evaluate changes in proteins and cellular processes in response to infection. The results showed that this approach is appropriate for the analysis of large proteomics datasets derived from different organisms in response to pathogen infection. The results demonstrated that networks of functionally interacting proteins can describe the complete set of host cell processes and biological pathways in response to pathogen infection. Furthermore, individual proteins were identified and validated that change the relative importance of different biological processes such as defense response to bacteria.

## Materials and methods

### Proteomics datasets from *A. phagocytophilum*-infected and uninfected ISE6 tick cells and HL60 human cells

The characterization of the host cell proteome in response to *Anaplasma phagocytophilum* infection was characterized in *Ixodes scapularis* tick vector embryo-derived cell line ISE6 (provided by U.G. Munderloh, University of Minnesota, USA) that serves as hemocyte model, and the human HL60 promyelocytic leukemia cells that serve as model of neutrophils (de la Fuente et al., 2005; Villar et al., 2015). The ISE6 cells were cultured in L-15B300 medium as previously described (Munderloh et al., 1994). HL-60 cells were cultivated in RPMI 1640 medium supplemented with 10% heat-inactivated fetal calf serum, 2 mM L-glutamine and 25 mM Hepes buffer as previously described (de la Fuente et al., 2005). Cells were infected with *A. phagocytophilum* (human isolate NY18) (Asanovich et al., 1997) as previously described (de la Fuente et al., 2005; Villar et al., 2015).

The proteomics dataset for tick cells was obtained from previously published results (Villar et al., 2015). Briefly, uninfected and infected tick cell cultures were sampled at 7 days post-infection. Total proteins were extracted, on gel concentrated, trypsin digested and analyzed by reverse phase liquid chromatography-tandem mass spectrometry (RP-LC-MS/MS) using an Easy-nLC II system coupled to an linear ion trap mass spectrometer model LTQ (Thermo Scientific). The MS/MS raw files were searched against a compiled database containing all sequences from Ixodida (77,195 Uniprot entries in March 2015) and Anaplasmataceae (64,677 Uniprot entries in March 2015) (http://www.uniprot.org) using the SEQUEST algorithm (Proteome Discoverer 1.4, Thermo Scientific). Searches were also performed against a decoy database in an integrated decoy approach. A false discovery rate (FDR) < 0.05 was considered as condition for successful peptide assignments and at least two peptides per protein were the necessary condition for protein identification. Three biological replicates were used for each of uninfected and infected tick cells. For the quantitative analysis of tick proteins, after discarding *Anaplasma* proteins in infected cells, the total number of peptide-spectrum matches (PSMs) for each tick protein was normalized against the total number of PSMs in tick cells and compared between control and infected cells by Chi2-test (*p* < 0.05).

For human cells, uninfected and infected human cell cultures (*n* = 2 independent cultures with ~10^6^ cells each) were sampled at 24 h post-infection. The cells were centrifuged at 10,000 × g for 5 min, resuspended in lysis buffer (100 mM Tris-HCl, 4% SDS, 50 mM DTT), boiled for 5 min and homogenized by passing through a needle (27 G). Samples were sonicated for 1 min in an ultrasonic cooled bath, followed by vortexing for 10 sec. After three cycles of sonication–vortex, total cell extracts were centrifuged at 200 × g for 5 min to remove cell debris. The supernatants were collected and protein concentration was determined using the RC DC Protein Assay (Bio-Rad, Hercules, CA, USA) with BSA as standard.

The protein extracts (200 μg) from uninfected and infected human cells were precipitated following the methanol/chloroform procedure^5^, resuspended in urea buffer (8 M urea, 50 mM ammonium bicarbonate, pH 8.8), reduced with 10 mM DTT for 1 h at 37°C and then alkylated with 50 mM iodoacetamide for 1 h at room temperature (RT) in darkness. The mixture was diluted four-fold to reduce urea concentration and then *in solution* digested overnight at 37°C with 60 ng/μl of sequencing grade trypsin (Promega) at 20:1 protein:trypsin (w/w) ratio in 50 mM ammonium bicarbonate, pH 8.8. Trifluoroacetic acid was added to a final concentration of 1% and the peptides were finally desalted onto OMIX Pipette tips C18 (Agilent Technologies), dried down, and stored at −20°C until mass spectrometry analysis.

The desalted protein digests was resuspended in 10% acetonitrile, 5% acetic acid in water and analyzed by RP-LC-MS/MS using an Ekspert nLC 415 system coupled to a 6,600 TripleTOF® mass spectrometer (AB SCIEX, Framingham, US) through Information-Dependent Acquisition (IDA) followed by SWATH (Sequential Windowed data independent Acquisition of the Total High-resolution Mass Spectra). Approximately 4 μg of each protein digest from each of the replicate samples were pooled together as a mixed sample for each condition (infected and uninfected human cells). Pooled mixed samples were then used for the generation of the reference spectral ion library as part of SWATH-MS analysis. The peptides were concentrated (on-line) using a 0.1 × 20 mm C18 RP precolumn (Thermo Scientific), and then separated using a 0.075 × 250 mm C18 RP column (New Objetive, Woburn, MA, USA) operating at 300 nl/min. Peptides were eluted using a 120 min gradient from 10 to 30% solvent B in solvent A followed by 10 min gradient from 30 to 40% solvent B in solvent A (Solvent A: 0.1% formic acid in water, solvent B: 0.1% formic acid in acetonitrile) and directly injected into the mass spectrometer for analysis. For IDA experiments, the mass spectrometer was set to scanning full spectra (390–1,400 m/z) using 250 ms accumulation time per spectrum, followed by up to 50 MS/MS scans (100–1,500 m/z). Candidate ions with a charge state between +2 and +5, and counts per second above a minimum threshold of 100, were isolated for fragmentation. One MS/MS spectrum was collected for 100 ms, before adding those precursor ions to the exclusion list for 15 sec (mass spectrometer operated by Analyst® TF 1.6, AB SCIEX). Dynamic background subtraction was turned off. MS/MS analyses were recorded in high sensitivity mode with rolling collision energy on and collision energy spread of 5. Two biological replicates were used for each of uninfected and infected human cells. For SWATH quantitative analysis, 6 μg of each independent sample were subjected to the cyclic data independent acquisition (DIA) of mass spectra using the SWATH variable windows calculator (V 1.0, AB SCIEX) and the SWATH acquisition method editor (AB SCIEX), following previously established methods (Gillet et al., 2012). A set of 50 overlapping windows was constructed (containing 1 m/z for the window overlap), covering the precursor mass range of 400–1,250 m/z. For these experiments, a 50 ms survey scan (390–1,400 m/z) was acquired at the beginning of each cycle, and SWATH MS/MS spectra were collected from 100 to 1,500 m/z for 70 ms at high sensitivity mode, resulting in a cycle time of 3.6 s. Collision energy for each window was determined according to the calculation for a charge +2 ion-centered upon the window with a collision energy spread of 15.

To create a spectral library of all the detectable peptides in the samples, the IDA MS raw files were combined and subjected to database searches in unison using ProteinPilot software v.5.0.1 (AB SCIEX) with the Paragon algorithm. Spectra identification was performed by searching against a complied database containing all the sequences from the *Homo sapiens* proteome and *A. phagocytophilum* taxonomy (Uniprot Databases: 70,939 and 21,847 entries, respectively in June 2017) with the following parameters: iodoacetamide cysteine alkylation, trypsin digestion, gel-based ID as special factor, identification focus on biological modification and thorough ID as search effort. The detected protein threshold was set at 0.05. An independent False Discovery Rate (FDR) analysis, using the target- decoy approach provided by ProteinPilot, was used to assess the quality of identifications. Positive identifications were considered when identified proteins reached a 1% global FDR. For SWATH processing, up to 10 peptides with seven transitions per protein were automatically selected by the SWATH Acquisition MicroApp 2.0 in the PeakView 2.2 software (AB SCIEX) with the following parameters: 15 ppm ion library tolerance, 5 min XIC extraction window, 0.01 Da XIC width, and considering only peptides with at least 99% confidence and excluding those which were shared or contained modifications. However, to ensure reliable quantitation, only proteins with 3 or more peptides available for quantitation were selected for XIC peak area extraction and exported for analysis in the MarkerView 1.3 software (AB SCIEX). Global normalization was performed according to the Total Area Sums of all detected proteins in the samples. A Student's *t*-test was used to perform two-sample comparisons between the averaged area sums of all the transitions derived for each protein across the two replicate runs for each sample under comparison, in order to identify proteins that were significantly differentially represented between infected and uninfected human cell samples.

Gene Ontology (GO) analysis for biological process was done by Blast2GO software (version 3.0; www.blast2go.com) (Villar et al., 2014). The mass spectrometry proteomics data have been deposited at the ProteomeXchange Consortium (http://proteomecentral.proteomexchange.org) via the PRoteomics IDEntifications (PRIDE) partner repository with the dataset identifier PXD002181, doi: 10.6019/PXD002181 and at the PeptideAtlas repository (www.peptideatlas.org) with the dataset identifier PASS01141, for tick and human cells, respectively. Supplementary Datasets 1 and 2 contain the complete list of tick and human proteins identified, data quantitation and GO annotations.

### Development of a network of proteins and cell processes in tick cells

A network is a set of nodes that are connected by edges. In the classic body of science devoted to food webs (Dunne et al., 2002) or parasitic networks (Lafferty et al., 2006) nodes are the interacting organisms, and links between nodes represent the strength with which they interact. The direction and strength of the interaction has a weight, which may be i.e., the number of times a parasite has been found on a host (Estrada-Peña et al., 2015).

The network construct proposed here is bipartite and metaphorical, meaning that a protein is the source node and the cell biological processes in which it is involved are the targets. Each protein was annotated with its role in a biological process according to gene ontology annotations. The network was built with a “source” (the protein) and a “destination” [the process(es) in which each protein is involved]. The link between source and destination has strength equivalent to the representation of the protein (expressed as normalized PSMs) in infected and uninfected cells. The Degree of each node was then calculated according to either the representation of the protein or the sum of links reaching a process (the destination).

The network is directed and the edge linking both nodes has a weight, which is the representation of the protein. Since the representation profile of each protein changes in either control or infected cells, it is therefore the indicator of the over- or under-representation of a protein, which radically changes the topology of the network, and therefore the relative importance of the processes. The WD, obtained from the sum of representation profiles of each link between proteins and processes is the fundamental brick of the network. All the calculations explained below were separately performed for the network of the sets of proteins detected in either uninfected (UtC) or infected cells (ItC).

All the work on the networks was done using the software Gephi 0.92 (www.gephi.org, accessed June 2016). First, clusters of each network were computed using the Louvaine algorithm. A cluster (also called the “modularity” of the network) is structurally cohesive to the extent that multiple independent relational paths among all pairs of members hold it together. The index detects groups of interacting nodes that are closer among them than with other nodes (Moody and Bevilacqua, 2003). In our framework, modularity has the implicit meaning of the set of proteins that are involved in a biological process more than in others. Clusters may have dozens of processes and hundreds of proteins, or consist only of few processes because proteins involved are not linked to other processes. Clusters or modules of the networks of proteins and processes of tick cells were considered here as biological pathways, and were named according to the process of the cluster with highest representation. This definition of “biological pathway” is different from typical pathways (metabolic, canonical signaling, etc.) and was adopted herein because the calculations using the Louvaine algorithm were strongly supportive of these clusters as “groups of proteins and processes that interact frequently among them.” Most important, these “pathways” changed in infected and uninfected cells, suggesting deep changes in the rewiring of the molecular machinery of the cells in response to infection.

We calculated the distribution of values of the WD of each node, which explains the assortativity of the network and its scale-free property, which strongly correlates with the network's robustness to failure. The exponent of a power-law function calculated the scale-free distribution and its significance, which demonstrates how close the distribution of WD is near the “perfect” power-law distribution, and therefore how fault tolerant the network is. The assortativity coefficient is the Pearson correlation (r) between the WD of nodes at both ends of each link in the network. The result ranges from −1 (low degree nodes often connect high degree nodes) to 1 (nodes of equal or similar degree are often connected). A significantly negative r means for disassortative networks, with strong hierarchical configurations, larger nodes connecting smaller nodes, as in scale-free networks. The positive correlation means for an assortative network, where hubs only link with other hubs (Rivas et al., 2012).

Centrality is a fundamental property of a network because it refers to nodes that connect high score nodes. Therefore, proteins with a high centrality can be regarded as pivotal parts of the network, while highly central processes can be considered as fundamental for the cell. We calculated the BNC, a measure of the importance of a node in the “traffic” between different nodes of a network, giving a higher score to a node that sits on many shortest path of other node pairs (Barthelemy, 2004). In our context, it is an indicator of “how central” is a process in the links between two proteins or other processes and the relative importance of a protein in the relationships of two or more processes. The rate of change of the BNC and WD between the networks built for infected and uninfected cells were calculated to capture the impact of the infection on the structure of the network. The objective was to focus on the detection of new proteins being represented, new processes operating, or significant changes in the indexes of the ItC when compared to UtC-derived networks.

### Development of a network of proteins and cell processes in human cells

In the case of proteins and processes detected in human cells, we adhered to the basic tenets described above for tick cells. We calculated the WD of each node from the representation profile of each protein linking a cell process. To evaluate the impact of the infection with *A. phagocytophilum*, we compared the rate of change of the WD of both represented proteins and cell processes (log transformed).

### RNAi for gene knockdown in tick cells

Primers were designed using the sequence of *I. scapularis* ras-related Rab14 protein B7QHS7 (GeneBank accession number XM_002414689) and selected for amplification a 273 bp region using oligonucleotide primers forward: 5′-CCCGCATCATCGAGGTGTGC-3′ and reverse: 5′-CCTCCTCGTATGTCACATCTC-3′. T7 promoter sequence was appended to the 5′ end of the forward and reverse primers for *in vitro* transcription and synthesis of dsRNA using the Megascript RNAi kit (Ambion, Austin, TX, USA). The unrelated Rs86 dsRNA was synthesized using the same methods described previously and used as negative control (Ayllón et al., 2013). The dsRNA was purified and quantified by spectrophotometry. RNAi experiments were conducted in cell cultures by incubating tick ISE6 cells with 10 μl dsRNA (5 × 10^10^-5 × 10^11^ molecules/μl) and 90 μl of fresh medium in 24-well plates using six wells per treatment. After 48 h of dsRNA exposure, medium containing dsRNA was removed and replaced with 1 ml fresh medium alone or containing cell free *A. phagocytophilum* NY18. Cells were incubated for 72 h, and then collected for DNA and RNA extraction and cell viability studies.

### RNAi for gene knockdown in human cells

siRNAs were obtained from GE Healthcare Dharmacon Inc. (Lafayette, CO, USA). Human Rab14 protein P61106 (GeneBank accession number NM_016322) was silenced using ON-TARGETplus SMARTpool Human *rab14* siRNA. As control, ON-TARGETplus Non-targeting Control Pool was used. In addition, Accell Red Non-targeting Control siRNA was used as transfection control to confirm the intake of siRNA molecules by human cells. Gene knockdown experiments were conducted by incubating human HL60 cells with siRNAs following manufacturer's recommendations, in 24-well plates using four wells per treatment. DharmaFECT (Dharmacon™) was added following manufacturer's recommendations to facilitate the transfection of HL60 cells. After 24 h of siRNA exposure, medium containing siRNA was removed and replaced with 0.5 ml fresh medium alone or containing cell free *A. phagocytophilum* NY18. Cells were incubated for 48 h, and then collected for DNA and RNA extraction and immunofluorescence microscopy assays.

### Determination of gene knockdown levels by real-time RT-PCR

Total RNA was extracted from UtC, ItC, UhC, and IhC using Tri Reagent (Sigma-Aldrich) following the manufacturer's recommendations and used to characterize the mRNA levels of selected genes. Real-time RT-PCR was performed on RNA samples using gene-specific oligonucleotide primers, the Kapa SYBR Fast One-Step qRT-PCR Kit (Sigma-Aldrich) and the QIAGEN Rotor-Gene Real-Time PCR Detection System (QIAGEN, Hilden, Germany). Oligonucleotide primers for analysis were as follows: (a) *I. scapularis* Rab14 forward: 5′-ACTCCCAACACGGTGATCTT-3′ and reverse: 5′-GGCTTCCGTCCTGAATGTTC-3′, (b) *H. sapiens* Rab14 forward: 5′-ATGGCAACTGCACCATACAAC-3′ and reverse: 5′-AGCTCCGTGTAACAGCCCTA-3′. A dissociation curve was run at the end of the reaction to ensure that only one amplicon was formed and that the amplicons denatured consistently in the same temperature range for every sample. The mRNA levels were normalized against tick *rps4* or human β*-actin* using the genNorm method [Delta-Delta-Ct (ddCt) method] as described previously (Ayllón et al., 2013). Normalized Ct values were compared between test dsRNA-treated tick cells and controls treated with Rs86 dsRNA or between UtC and ItC by Student's *t*-test with unequal variance (*p* = 0.05; *n* = 6 biological replicates). In the case of human cells, normalized Ct values were compared between test siRNA-treated and control cells treated with Non-targeting pool siRNA or between UhC and IhC by Student's *t*-test with unequal variance (*p* < 0.05; *n* = 4 biological replicates).

### Determination of *A. phagocytophilum* DNA levels by real time PCR

Total DNA was extracted from infected cells using a NucleoSpin Tissue kit (Macherey-Nagel GmbH and Co., Duren, Germany). DNA samples were analyzed by real-time PCR using gene-specific primers for *A. phagocytophilum msp4* as previously described (Ayllón et al., 2013). Normalized Ct values were compared between dsRNA/siRNA-treated tick cells and controls treated with Rs86 dsRNA or Non-targeting pool siRNA by Student's *t*-test with unequal variance (*p* < 0.05; *n* = 4–6 biological replicates).

### Immunofluorescence microscopy assays

Uninfected and *A. phagocytophilum*-infected HL60 cell slides were prepared using a cytocentrifuge. The slides were dried and fixed immediately with acetone for 10 min at RT, blocked by adding 50 μl of 5% BSA diluted in PBS, and incubated in a humidified chamber at RT for 1 h. After 3 washes in 0.05% Tween 20/PBS (wash buffer), the cells were incubated with rabbit polyclonal anti-*A. phagocytophilum msp4* antibodies (Contreras et al., 2017) diluted 1:100 in PBS for 1 h at RT. After additional washes, the cells were incubated with 100 μl of FITC conjugated goat anti-rabbit IgG secondary antibodies (Sigma-Aldrich, St. Louis, MO, USA), diluted 1:200 in PBS, for 1 h at RT. Finally, the slides were mounted using Prolong Gold antifade reagent with DAPI reagent (Molecular Probes, Eugene, OR, USA). To confirm the intake of siRNA molecules by human cells, cytocentrifuge preparations of HL60 cells treated with Accell Red Non-targeting Control siRNA were prepared and then mounted as described above. The slides were examined using a Zeiss LSM 800 laser scanning confocal microscope (Carl Zeiss, Oberkochen, Germany).

### Annexin V-FITC staining to detect tick cell viability

Approximately 5 × 10^5^ uninfected and *A. phagocytophilum*-infected tick ISE6 cells were collected after different treatments. Apoptosis was measured by flow cytometry using the Annexin V-fluorescein isothiocyanate (FITC) apoptosis detection kit (Immunostep, Salamanca, Spain) following the manufacturer's protocols. The technique detects changes in phospholipid symmetry analyzed by measuring Annexin V (labeled with FITC) binding to phosphatidylserine, which is exposed in the external surface of the cell membrane in apoptotic cells. Cells were stained simultaneously with the non-vital dye propidium iodide (PI) allowing the discrimination of intact cells (Annexin V-FITC negative, PI negative) and early apoptotic cells (Annexin V-FITC positive, PI negative). All samples were analyzed on a FAC-Scalibur flow cytometer equipped with CellQuest Pro software (BD Bio-Sciences, Madrid, Spain). The viable cell population was gated according to forward-scatter and side-scatter parameters. The percentage of apoptotic, dead, necrotic and viable cells was determined by flow cytometry after Annexin V-FITC and PI labeling and compared between both test and control dsRNA treated infected and uninfected cells by Student's *t*-test with unequal variance (*p* < 0.05; *n* = 6 biological replicates).

## Results

### Development of a graph approach for the characterization of networks of proteins and cell processes in response to *A. phagocytophilum* infection of tick vector and human cells

**Figure 1:**
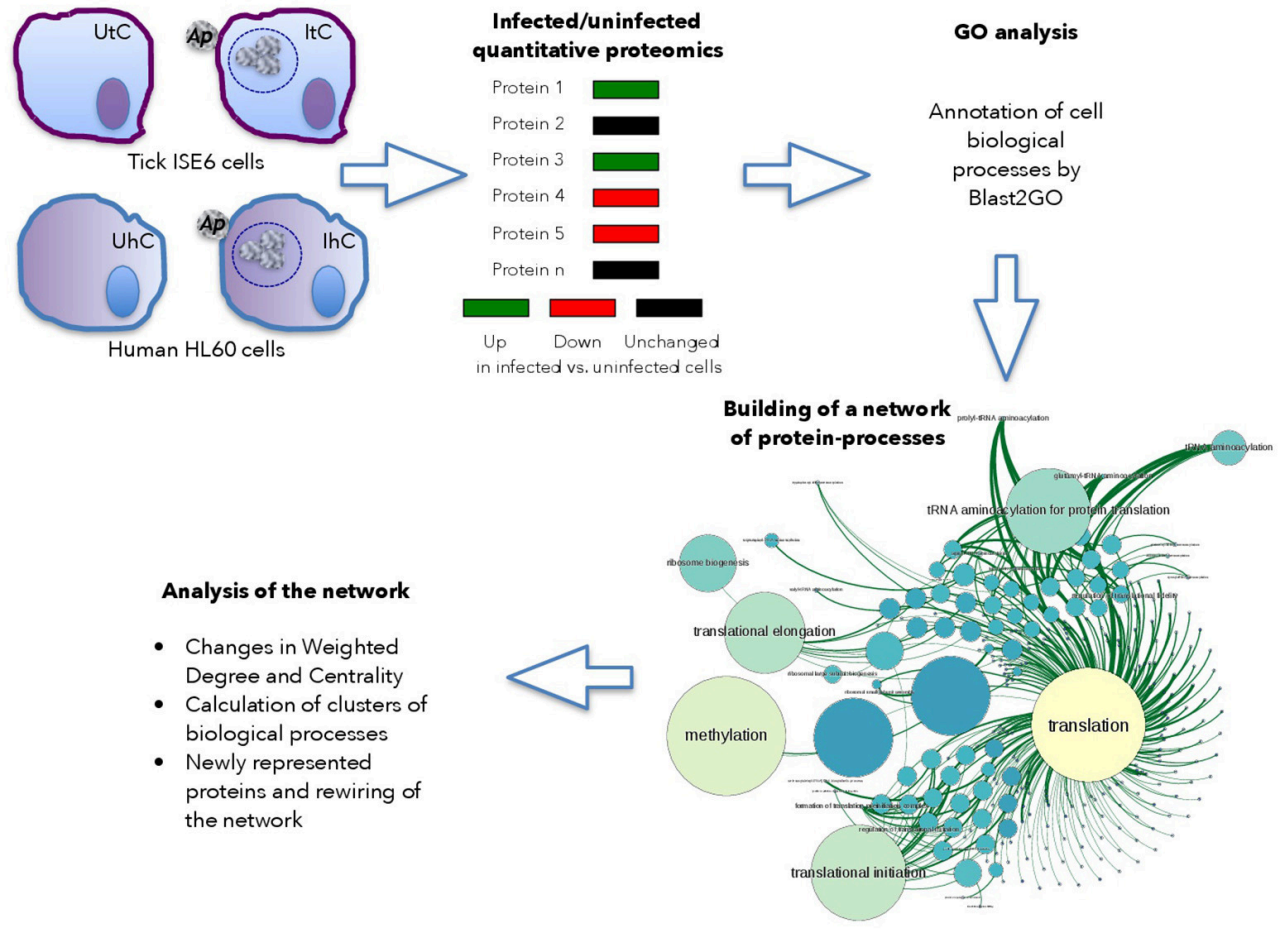
Use of graph theory to characterize tick and human cell protein response to infection. Schematic representation of the framework used for analysis.

The host cell proteome in response to *A. phagocytophilum* infection was characterized in *I. scapularis* tick vector ISE6 cells that serve as hemocyte model (Supplementary Dataset 1), and human HL60 promyelocytic leukemia cells that serve as model of neutrophils (Supplementary Dataset 2; de la Fuente et al., 2005; Villar et al., 2015). Proteins from uninfected and infected cells were analyzed by reverse phase liquid chromatography-tandem mass spectrometry (RP-LC-MS/MS) using an Easy-nLC II system coupled to a linear ion trap mass spectrometer model LTQ (Thermo Scientific, San Jose, CA, USA) or an Ekspert nLC 415 system coupled to a 6600 TripleTOF® mass spectrometer (AB SCIEX, Framingham, USA) for tick and human cells, respectively. Three and two biological replicates for tick and human cells, respectively were used for each uninfected and infected cells. For the quantitative analysis of tick proteins, after discarding *Anaplasma* proteins in infected cells, the total number of peptide-spectrum matches (PSMs) for each tick protein was normalized against the total number of PSMs in tick cells (Figure 1). For the quantitative analysis of human proteins, the total area sums for each protein were normalized against the total area sums of all detected proteins in human cells (Figure 1). Gene ontology (GO) analysis for biological process was done by Blast2GO software (version 3.0; www.blast2go.com) (Villar et al., 2015; Figure 1). In our approach, the network is composed of nodes (proteins) influencing next level successive nodes (cell processes) (Figure 1). In the graph approach, the functional protein-protein interactions have a weighted degree on one or several cell processes, which in our application is the representation profile of the protein (Figure 1). This is the basis for the Centrality of the networks of proteins and cell processes detected in either uninfected or infected cells. Centrality is a property of the networks that refers to nodes that are fundamental for the cohesion of the network (Chisanga et al., 2017). Therefore, proteins with a high centrality are pivotal parts of the network, while highly central processes can be considered as fundamental for the cell response to infection.

### Simple indexes define the network of protein processes in tick ISE6 cells, a model of vector hemocytes

In the tick cell model, the BNC (Gago, 2014) that represents the importance of the vertex in the network was calculated. The change rate of the BNC was calculated between uninfected (UtC) and infected (ItC) tick cells to characterize the response to infection in the structure of the network of proteins and cell processes. The analysis was focused on the detection of newly represented proteins, newly operating processes, and/or significant changes in the indexes of the ItC-derived when compared to UtC-derived networks. The network of proteins and processes in UtC showed 7,595 nodes, which were connected by a total of 11,326 links. The network of ItC had 7,520 nodes and 11,268 links. A total of 6,812 proteins involved in 784 processes were identified in UtC, while ItC showed 6,745 proteins and 783 processes (Supplementary Dataset 3). The infection of tick cells with *A. phagocytophilum* resulted in changes in protein levels and the appearance of proteins not present in UtC. A total of 671 and 605 proteins were unique to UtC and ItC, respectively, and 22 and 21 processes were unique to UtC and ItC, respectively (Table 1).

**Table 1:** Number of tick cell proteins and processes on each biological pathway, including those that are unique to UtC and ItC (in parenthesis).

**Biological pathway**	**UtC**		**ItC**	
	**Proteins**	**Processes**	**Proteins**	**Processes**
Metabolism	977 (149)	33 (3)	989 (130)	36 (6)
Redox	734 (67)	115 (3)	724 (57)	116 (4)
Proteolysis	667 (72)	44 (0)	661 (66)	45 (1)
Signal processing	249 (19)	52 (2)	241 (11)	53 (3)
Transport	372 (46)	84 (2)	355 (29)	82 (0)
Phosphorylation	374 (30)	101 (0)	373 (29)	109 (8)
Translation	241 (29)	28 (0)	229 (17)	28 (0)
Transcription	310 (33)	77 (2)	303 (26)	76 (1)

Two indexes based on the WD demonstrated that both ItC and UtC networks have properties of natural, not random, highly hierarchical networks. The WD of all nodes in both UtC and ItC networks followed a power-law distribution with an exponent of 2.60 for UtC (*p* = 0.039) and 2.38 for ItC (*p* = 0.004), lower *p*-values meaning a stronger similarity to the scale-free distribution, which is a fundamental property of natural networks. It is interesting to note that *A. phagocytophilum* infection did not change the scale-free property of WD, therefore not collapsing the metabolism of the tick. The assortativity coefficient (r) of both UtC abd ItC networks, which is a measure of its hierarchical configuration was *r* = −0.2477 (UtC) and *r* = −0.2468 (ItC). Therefore, both UtC and ItC networks were dissortative (r index was significantly negative), indicating a strong hierarchical configuration that shows high resilience to random protein removal.

### Tick cell biological pathways are unambiguously defined by indexes of centrality

The clusters of each network were computed as the set of proteins and cell processes that interact more among them than with others, using the Louvaine algorithm as implemented in the software Gephi 0.92 (www.gephi.org, accessed June 2016). In our analysis, this concept of modularity has the implicit meaning of proteins that are involved in certain cell processes more than in others, and groups of cell processes that are triggered by specific groups of proteins. The components (proteins and processes) in each cluster are thus the biological pathways that are cohesively connected.

The detection of clusters resulted in 146 and 141 biological pathways in UtC and ItC, respectively. Most of them were tightly connected in the network, since many proteins were involved in processes belonging to different biological pathways. This effect produced a main giant component with some isolated biological pathways that were not connected to that main core of the network (Supplementary Figures 1, 2). The UtC and ItC networks showed 116 and 114 connected biological pathways, respectively, with 21% of the biological pathways isolated among them and unconnected with the main core of the network. Between all detected biological pathways, the first eight contained 75.9% of the nodes and 79.8% of the links in UtC, and the 77.1% of the nodes and 81% of the links in ItC. The most relevant biological pathways were metabolism, redox, proteolysis, signal processing, transport, phosphorylation, translation, and transcription (including DNA and RNA-related processes) (Table 1).

### *A. phagocytophilum* infection rewires the network of tick vector cell processes and changes the relative importance of some biological pathways

**Figure 2:**
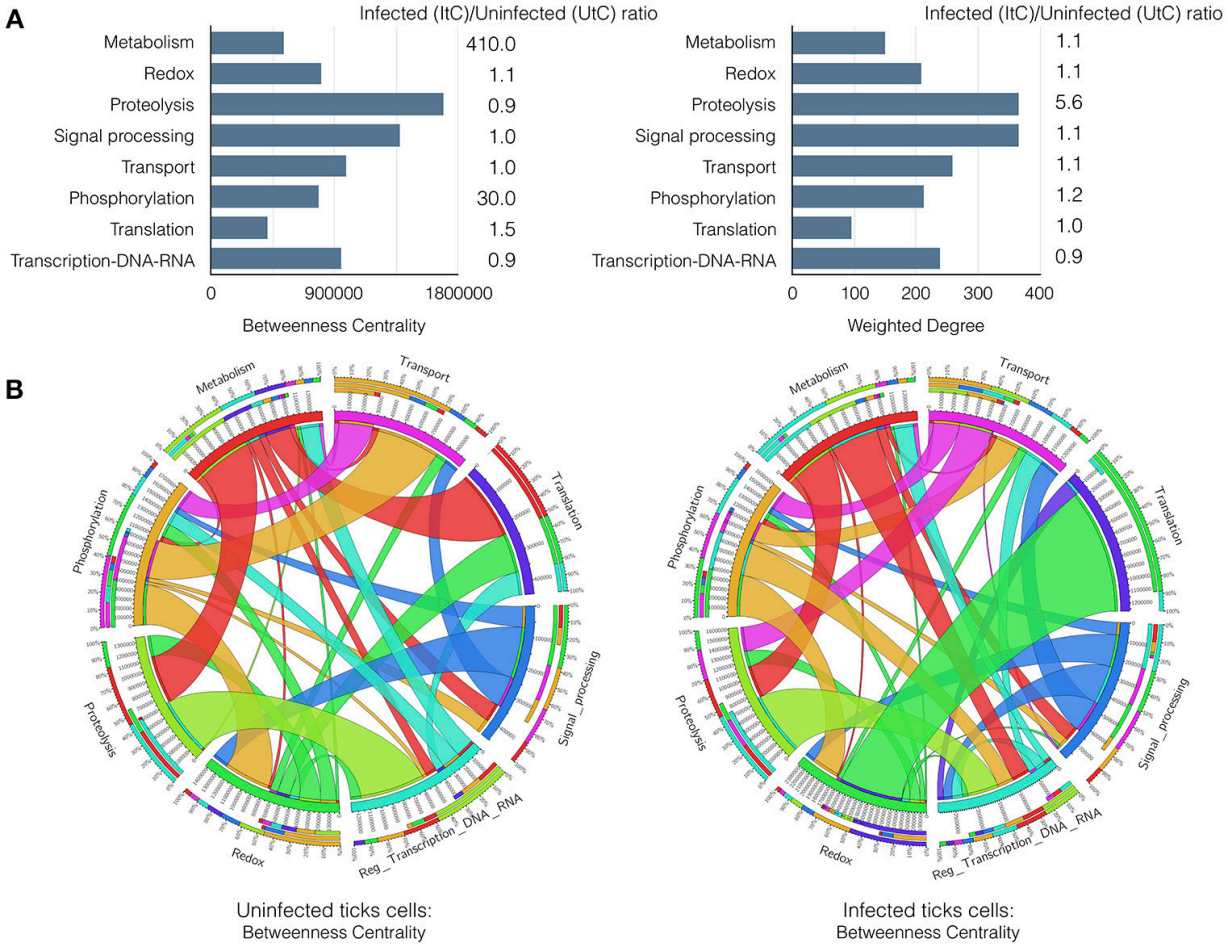
Results of the graph analysis in *A. phagocytophilum* infected and uninfected tick vector cells. **(A)** Variations in Betweenness Centrality (BNC) and Weighted Degree (WD) values between UtC and ItC. Bars represent BNC and WD values for UtC. The fold change between ItC and UtC is included near each bar. **(B)** Comparison of the WD between UtC and ItC for the proteins of each biological pathway and their connections to other biological pathways. A circle layout displaying the relationships between biological pathways and proteins linking them in both UtC and ItC is shown. Each sector of the circle shows the names of the eight most important biological pathways detected by a clustering algorithm. The first sector below the name shows the percentage of proteins of that biological pathway present in other biological pathways. The second sector indicates the percentage of proteins belonging to other biological pathways that also affect the biological pathway of reference. The third sector displays the percentage of proteins that belong to that biological pathway and are also involved in other biological pathways. The fourth sector displays the actual value of the parameter displayed in the figure. Colors for these three sectors indicate the set of proteins from/to different biological pathways that are shared. The white band above the fourth sector indicates the proportion of “in-coming proteins” while the rest of the sector is used to display the proportion of “out-going proteins”. The relative size of each band is proportional to the WD of the proteins shared among the biological pathways. Note: The Supplementary Figure 3 displays an example with annotations and labels to interpret the circle and band layouts.

An explicit comparison of the BNC and WD values between UtC and ItC networks showed changes in relevant cell processes and the resulting contribution to each biological pathway (Figure 2A). The results showed that in UtC, proteolysis had the highest representation in the network, which together with signal processing and transcription were the most central biological pathways, thus linking the most relevant cell processes. The changes in ItC resulted in a 410- and 30-fold increase in BNC of metabolism and phosphorylation, respectively. Proteolysis showed slight changes in centrality but increased in more than 5-fold the WD. These results suggested an increase in the number and/or representation of proteins affecting proteolysis in response to infection, even if this biological pathway did not change its relative position in Centrality. However, both metabolism and phosphorylation became highly central in ItC, with only a slight increase in WD.

The most relevant changes were observed in the rewiring of the proteins involved in the biological pathways, because each protein belongs to a cell process but is also secondarily involved in biological pathways belonging to other processes. It was possible to evaluate how proteins belonging to a biological pathway also affected other biological pathways in either UtC or ItC (Figure 2B). Changes in UtC when compared to ItC showed that (i) a set of proteins involved in both transport and proteolysis were absent in UtC but present in ItC, (ii) the combined WG of proteins in translation-redox in ItC was only 10%, increasing to more than 80% in UtC, (iii) more than 50% of WG of proteins in transcription were also involved in translation in ItC, but were absent in UtC, and (iv) connections between signal processing and transcription in ItC were not apparent in UtC (Figure 2B and Supplementary Figure 3).

### Tick and human cells respond differently to infection with *A. phagocytophilum*

**Figure 3:**
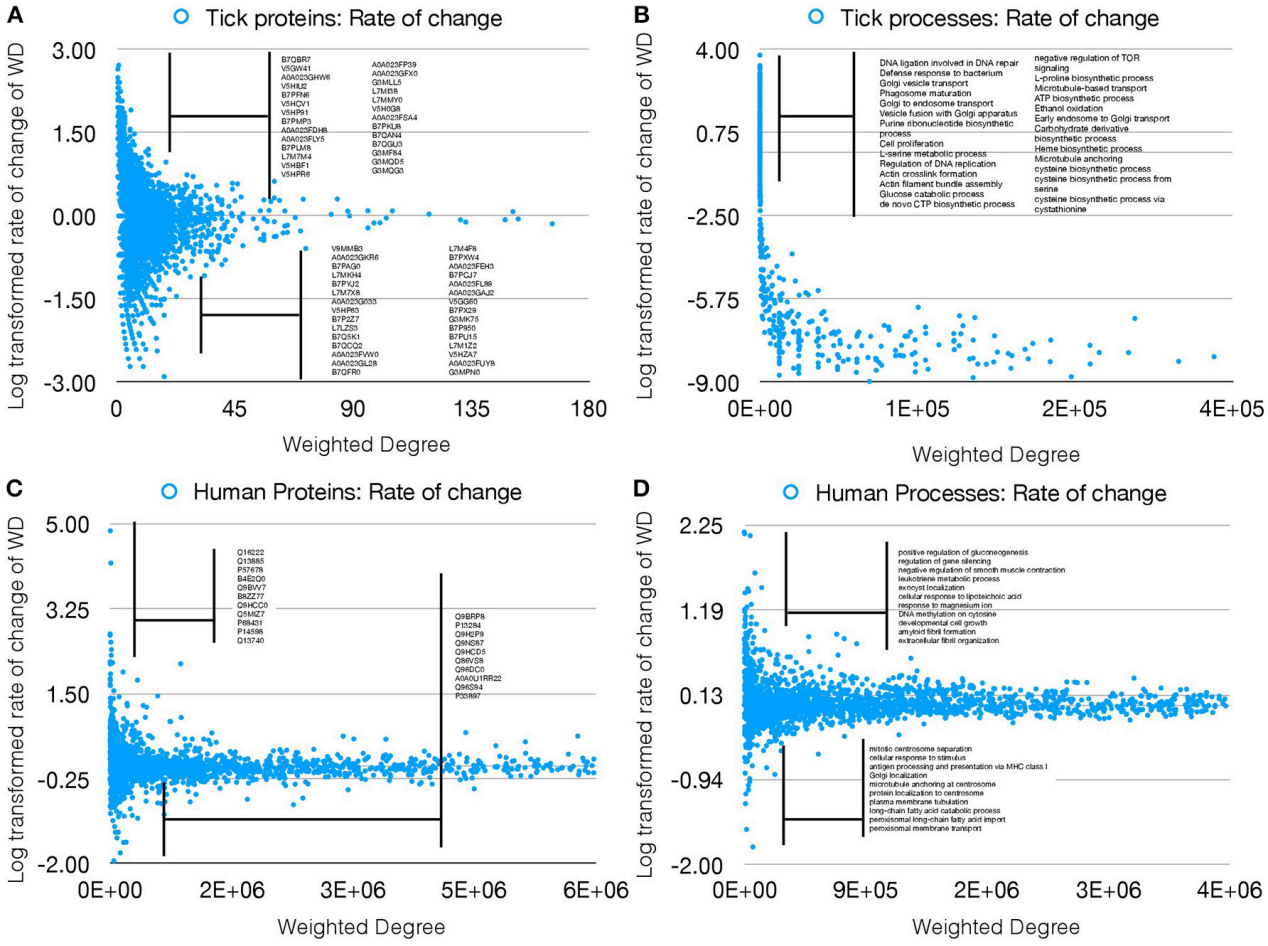
Rate of WD change of tick and human cell protein and processes. **(A)** The rate of WD change of the proteins in tick vector cells. The WD of the proteins in UtC is shown, together with the log-transformed rate of change in ItC. Most highly over- and under-represented proteins are shown (3- and 5-fold for tick and human cells, respectively). Proteins represented only in UtC or ItC were not included. **(B)** The rate of change of WD of the cell processes in tick vector cells. The WD of the cell processes in ItC is shown, together with the log-transformed rate of change in ItC. Most highly over- or under-represented cell processes are shown. Processes appearing only in UtC or ItC were not included. **(C)** The rate of change of WD of the proteins in human cells. The WD of the proteins in UhC is shown, together with the log-transformed rate of change in IhC. Most highly over- and under-represented proteins are shown. **(D)** The rate of change of WD of the cell processes in human cells. The WD of the cell processes in IhC is shown, together with the log-transformed rate of change in IhC. Most highly over- and under-represented cell processes are shown.

The properties of the networks for tick proteome were supported by the presence/absence of several proteins in either UtC or ItC, deeply impacting the network topology and capturing the impact of pathogen infection on the tick cell response. We then proceeded to compare the changes in annotated proteins of *A. phagocytophilum* infected (IhC) and uninfected (UhC) human HL60 cells. The analysis was focused on the rate of change of WD as the measure of changes in the networks, to evaluate if only this index could represent changes in both proteins and cell processes in response to infection. The rate of change of WD was calculated for each protein and cell process in both infected and uninfected tick and human cells, and log transformed to produce comparable results (Figures 3A–D).

The results showed that the WD change rate was higher for tick (Figure 3B) than human cell processes (Figure 3D), but fewer over- and under-represented processes were present in human when compared to tick cells (Figures 3B,D), demonstrating a higher impact of *A. phagocytophilum* infection on tick cells. Similarly, more over- and under-represented proteins were present in ItC than in IhC (Figures 3A,C). However, the WD change rate was higher in IhC than in ItC for identified over-represented proteins. The biological significance of these results is that *A. phagocytophilum* infection had a higher impact on tick cells, but the fewer human proteins manipulated by the pathogen were more highly differentially represented.

### Detection of tick vector and human host proteins playing a major role in metabolism changes in response to *A. phagocytophilum* infection

The network constructed with tick proteomics data was used to identify individual proteins playing a major role in response to infection with *A. phagocytophilum*. The characterization of the changes in the metabolism of both UtC and ItC showed a variation in the centrality of defense response to bacteria, evasion or tolerance of host defense response, hippo signaling, glucose catabolic process, cellular glucose homeostasis, and regulation of apoptotic process (Supplementary Table 2). These processes have been implicated in tick-*A. phagocytophilum* interactions using a systems biology approach (Sultana et al., 2010; Severo et al., 2013; Ayllón et al., 2015; de la Fuente et al., 2015, 2016; Villar et al., 2015; Cabezas-Cruz et al., 2016, 2017; Gulia-Nuss et al., 2016; Shaw et al., 2017). The increase in the centrality of these processes was driven by changes in the WD of certain proteins that were only represented in either ItC or UtC or over-represented in ItC (Supplementary Table 2). Furthermore, some of the proteins represented only in ItC, linked the defense response to bacteria with other processes such as phagosome maturation, killing of cells of the organisms, innate immune response, endocytic recycling, and small GTPase mediated signal transport, which are all involved in tick cell response to infection (Sultana et al., 2010; Severo et al., 2013; Ayllón et al., 2015; de la Fuente et al., 2015, 2016; Cabezas-Cruz et al., 2016, 2017; Gulia-Nuss et al., 2016; Shaw et al., 2017). Focusing on the defense response to bacteria, which Centrality increased by 53,000-fold in response to infection, the proteins driving this change were the ras-related proteins Rab14 (B7QHS7 and L7M7N3) over-represented in ItC, and the antimicrobial peptide microplusin (Q09JR4) that was represented only in ItC (Supplementary Table 2). Of these proteins, ras-related proteins were selected for functional analysis by gene knockdown because these proteins are highly evolutionary conserved (Supplementary Figure 4), and function as binary molecular switches that control intracellular signaling networks affecting processes that have been shown before to be involved in tick cell infection by *A. phagocytophilum* (Sultana et al., 2010; Severo et al., 2013; Ayllón et al., 2015; de la Fuente et al., 2015, 2016; Villar et al., 2015; Cabezas-Cruz et al., 2016, 2017; Gulia-Nuss et al., 2016; Shaw et al., 2017). The ras-signaling pathway includes processes such as cell defense and survival, actin cytoskeletal integrity, cell proliferation, cell differentiation, cell adhesion, transcription, and gene expression, apoptosis, endocytosis, and cell migration (McCormick, 1995; Kuijl and Neefjes, 2009). In particular, ras-related proteins may be activated by intracellular bacteria on phagosomal membranes to prevent the transfer of bacteria from phagosomes to lysosomes to favor pathogen survival (Kuijl et al., 2007; Kuijl and Neefjes, 2009). *A. phagocytophilum* actively modifies its host cell-derived vacuole (Huang et al., 2010a,b). Furthermore, *A. phagocytophilum* is known to hijack Rab10 and other endoplasmic reticulum membrane markers to its vacuole to complete the infection cycle in both vertebrate and tick host cells (Huang et al., 2010a,b; Truchan et al., 2016a,b). In contrast to tick cells, in human cells the ortholog for the tick ras-related protein Rab14 B7QHS7 (P61106) was under-represented in IhC when compared to UhC. The human ras-related protein Rab14 was also selected for functional analysis by gene knockdown to compare tick and human cell response to infection.

**Figure 4:**
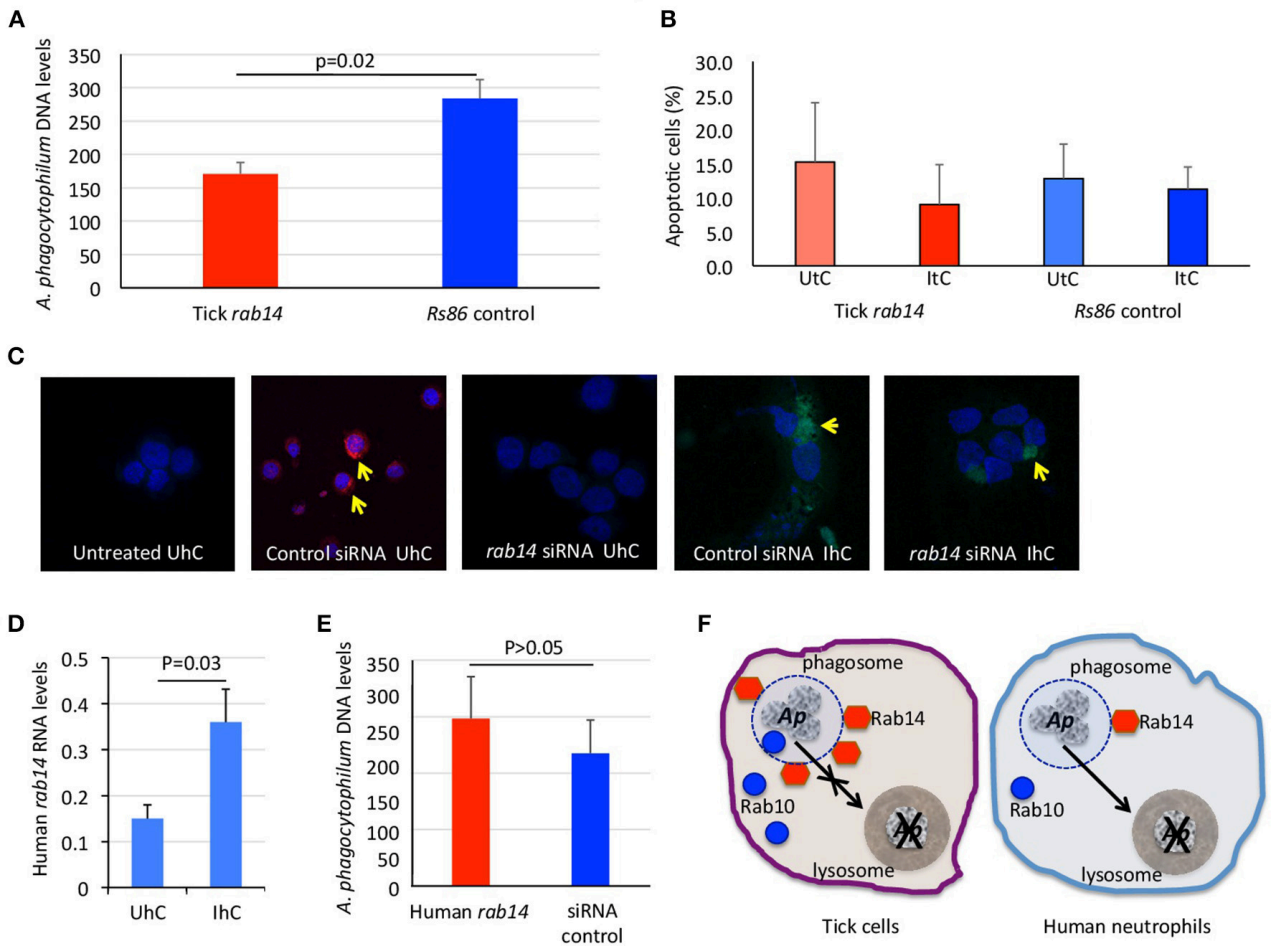
Functional analysis by RNAi supports a role for tick and human Rab14 in *A. phagocytophilum* infection of host cells. **(A)** A 75–83% knockdown by RNAi of tick *rab14* (B7QHS7) in tick cells resulted in a 40% decrease in *A. phagocytophilum* infection levels, suggesting that *A. phagocytophilum* increases the levels of Rab14 to facilitate infection. Tick ISE6 cells were treated with *rab14* dsRNA and control cells were treated with the unrelated *Rs86* dsRNA. DNA samples from infected cells were analyzed by real-time PCR using the *A. phagocytophilum* major surface protein 4 (*msp4*) gene-specific primers. Normalized Ct values were compared between groups by Student's *t*-test with unequal variance (*p* = 0.02; *n* = 6 biological replicates). **(B)** Tick *rab14* knockdown did not affect cell viability. The percent of apoptotic tick ISE6 cells was determined after RNAi with *rab14* test and *Rs86* control dsRNAs by flow cytometry using the Annexin V-fluorescein isothiocyanate (FITC) apoptosis detection kit. The percentage of apoptotic cells was compared between both test and control dsRNA treated UtC and ItC by Student's *t*-test with unequal variance (*p* > 0.05; *n* = 6 biological replicates). **(C)** Representative images of immunofluorescence analysis of UhC and IhC incubated with either ON-TARGETplus SMARTpool Human *rab14* siRNA or control ON-TARGETplus Non-targeting Control Pool siRNA. Cells were stained with rabbit anti-*A. phagocytophilum msp4* antibodies, labeled with FITC (green, arrows) and DAPI (blue). To confirm the uptake of siRNA, cells were treated with Accell Red Non-targeting Control siRNA (red, arrows) and labeled with DAPI (blue). **(D)** Human *rab14* was up-regulated at the mRNA level in response to infection. The RNA levels of human *rab14* (P61106) were determined by real-time RT-PCR in UhC and IhC. Normalized Ct values were compared between groups by Student's *t*-test with unequal variance (*p* = 0.03; *n* = 4 biological replicates). **(E)** A 31–52% knockdown by RNAi of *rab14* in human HL60 cells did not affect *A. phagocytophilum* infection levels, suggesting that Rab14 protein levels decrease post-transcriptionally in human neutrophils to control *A. phagocytophilum* infection. Human HL60 cells were treated with *rab14* siRNA or control ON-TARGETplus Non-targeting Control Pool siRNA. DNA samples from infected cells were analyzed by real-time PCR using the *A. phagocytophilum* major surface protein 4 (*msp4*) gene-specific primers. Normalized Ct values were compared between groups by Student's *t*-test with unequal variance (non-significant, *p* > 0.05; *n* = 4 biological replicates). **(F)** Proposed model of ras-related protein function in *A. phagocytophilum*-infected tick and human cells. In tick cells, *A. phagocytophilum* (*Ap*) increases the levels of active ras-related proteins Rab14 in phagosomal membranes to prevent the transfer of bacteria from phagosomes to lysosomes and hijacks Rab10 and other endoplasmic reticulum membrane proteins to its vacuole to complete the infection cycle and favor pathogen survival and facilitate infection. In human neutrophils, the decrease in Rab10 levels appears as a post-transcriptional mechanism to control *A. phagocytophilum* infection.

The results of *ras-related proteins rab14* B7QHS7 and P61106 gene knockdown by RNA interference (RNAi) in tick and human cells, respectively corroborated that these proteins play a role during infection without affecting tick cell viability (Figures 4A–C). The results suggested that *A. phagocytophilum* increases the levels of Rab14 in ticks to facilitate infection while in human neutrophils the decrease in Rab14 protein levels appears as a post-transcriptional mechanism to control *A. phagocytophilum* infection (Figures 4D–F).

## Discussion

A method based on the graph theory with broad application in social and ecological relationships among interacting organisms (Jordano et al., 2006; Kourtellis et al., 2013) was applied to unambiguously detect the changes produced by *A. phagocytophilum* infection on the proteome of tick vector and human host cells. This is the first application of graph theory to a dataset of annotated proteins from which we derived a comprehensive view of biological pathways, the relative importance of each protein and its place in the context of the proteome in response to infection. This methodological approach was applied to two different hosts, further supporting its application to the characterization of cell response to different stimuli in model and non-model organisms. Furthermore, the method was successfully applied to results obtained from two different proteomics approaches better adapted to model (human) and non-model (tick) organisms, therefore demonstrating its validity for the analysis of different proteomics datasets.

Our method was based on previous reports using classical approaches to the study of cell response to pathogen infection by comparing different gene expression and/or protein representation rates (Villar et al., 2015). However, our approach intended to capture the complete landscape of the cell biological machinery, and how the different representation of proteins act together to up- or under-regulate the cellular processes. We used uninfected and infected tick cells only as a proof of concept, but the network framework could be built using any set of annotated proteins. The method developed here and applied to the cell response to *A. phagocytophilum* infection captured many proteins that remained undetected using the classical approach based on its rate of change. The network analysis captured the individual significance of many proteins, even if not statistically significantly changed in response to infection, and evaluated their combined actions on the target process(es). Therefore, the advantage of this method lays on its ability to integrate the groups of proteins and their combined actions on specific biological processes, allowing understanding the complete cell response to a stimuli.

The results demonstrated that changes in the tick proteome were driven by modifications in protein representation in response to *A. phagocytophilum* infection. The conversion of scores from large datasets of annotated proteins into a graph structure allowed the tractability of cell processes into biological pathways. We demonstrated that the distribution of WD values of the networks has the properties expected in a natural network, and that the framework has a biological significance after clustering cell processes into communities of biological pathways. The method has been widely used for the analysis of different interacting entities (Chautard et al., 2009), and is therefore supported by a large coherent corpus of science (Perc et al., 2013).

Pioneering efforts focused on the gene-gene co-expression using a molecular interaction network (Poirel et al., 2013) demonstrated that co-expressed genes should participate in coherent network structures. Vinayagam et al. (2014) addressed the physical interactions between proteins (activation-inhibition relationships) through the use of a network describing relationships between proteins, without providing insights on cell processes. A similar network of interactions was built for 70 viral open reading frames (vORFs) interacting with 579 proteins targeted by the vORFs (Pichlmair et al., 2012). However, the interactions were measured from the visual topology of the network, without characterizing indexes for interactions. Previous studies showed the importance of WD in describing metabolic networks (Ma and Zeng, 2003; Zhang and Horvath, 2005), demonstrating the emergence of a power-law distribution (scale free topology) intimately linked to the network of gene co-expression. Our results using proteomics datasets corroborated that WD follows a power-law distribution at the protein/cell processes level, which is of extreme importance for the resilience of the complete network.

The *A. phagocytophilum* infection had a higher impact on tick than human proteome. Since most proteins were linked to several cell processes, the changes in protein representation affected simultaneously different biological pathways. The method allowed discerning cell processes that were affected by pathogen infection from those that remained unaffected. The deep subversion of the tick proteome by *A. phagocytophilum* probably reflects ancient ecological tick-pathogen associations (de la Fuente et al., 2015). Ticks not only tolerate infection by *A. phagocytophilum*, but infected ticks have higher adaptation, survival and vector capacity (de la Fuente et al., 2017). In contrast with the infection of several cell types during *A. phagocytophilum* life cycle in ticks, pathogen infects only neutrophils in vertebrate hosts, and therefore subtle changes in the proteome should be expected (de la Fuente et al., 2005; Ayllón et al., 2015; Severo et al., 2015; Villar et al., 2015). The results supported that human neutrophils but not tick cells limit pathogen infection through differential representation of ras-related proteins.

The method has however some gaps. The absence of a completely annotated proteome as such currently available in ticks and other organisms would provide a partial view of the cell biological processes. For a partially annotated proteome it is not possible to include a protein in the network if the target process is not defined, and in consequence the centrality indexes of the processes would be affected. In contrast, for fully annotated proteomes as occurs for humans and model organisms, network analyses may collapse and require alternative approaches as used here for the analysis of human cell response to infection. These developments warrant further research to better understand cell response to stimuli such as pathogen infection.

## Conclusions

Approaches in this field have been commonly restricted to gene-gene or protein-protein interactions, without evaluating the metabolic signal that emanates from these interactions. Our approach addressed the major challenge of integrating information switching from static to dynamic interaction networks, revealing the nature of changes in the proteome and the resulting cell processes and biological pathways. The metaphorical network linking proteins with cell processes revealed the design principles of the metabolic organization. The application of this method identified unexpected roles of proteins after cell infection, showing the intimate mechanisms of metabolic rewiring, and has wide applications in other models. Therefore, this methodological approach could be applied to other host-pathogen models to identify host derived key proteins in response to infection that may be used to develop novel control strategies for arthropod-borne pathogens.

## Author contributions

AE-P, MV, and JF conceived the study and designed the experiments. MV, SA-J, VL, and PA performed the experiments. AE-P, MV, AC-C, and JF performed data analysis. AE-P, MV, and JF wrote the manuscript. All authors approved and contributed to the final version of the manuscript.

### Conflict of interest statement

The authors declare that the research was conducted in the absence of any commercial or financial relationships that could be construed as a potential conflict of interest.
