# Supplementary material for: Use of Graph Theory to Characterize Human and Arthropod Vector Cell Protein Response to Infection With Anaplasma phagocytophilum
Source: Front Cell Infect Microbiol. 2018 Aug 3;8:265. doi: 10.3389/fcimb.2018.00265 (PMC6086010; doi:10.3389/fcimb.2018.00265)
Supplement: Supplementary file 1 [file Table_1.PDF]

Supplementary Table 1. Terms and it formal descriptions and classic usage in the theory of networks, and its interpretation in the biological context used in our method.

**Term**	**Description**	**Classic applications**	**Biological meaning**
Network	A set of nodes and links that describe interactions. Nodes may be any kind of objects and links can describe any kind of interactions.	Networks have wide applications in the field of food-webs, parasitic or commensalist relationships between organisms. They are widely used in describing social networks	Nodes are proteins with attributes (i.e. representation profiles). The annotation of the protein produces a virtual target node. Then the classic "source-target" structure of a network is produced.
Variables	Each node and each link are loaded with variables that affect the structure of the network and describe it.	Nodes and links are loaded with data on i.e. number of interactions between two organisms. The rest of indexes are obtained from these basic measures of interactions.	The only variable is carried by the node of each protein, and corresponds to its representation profile. Target nodes (processes) receive links from several proteins and are therefore loaded with variables "received" from the proteins pointing to that process.
Weighted Degree (WD)	A measure of the number of links between two nodes weighted by the total number of links.	In parasite-host networks, WD is obtained by the number of records of a parasite in a host, times the number of parasites per host individual	WD for source nodes (proteins) is the representation profile. WD for target nodes (processes) is obtained by the number of proteins linked to that process, times the representation profile of each protein.
Communities	Group of nodes that are more connected among them than with other nodes of the complete network.	In parasite-host networks, several host species may be preferred by some parasites, while others are hosts of a different set of parasites. Communities reflect the groups of interacting	Nodes or either proteins or processes that are more connected among them that with other proteins or processes. The method detects groups of processes that act together, and the proteins that drive these processes. Communities are herein interpreted as biological pathways.

		species.	
Centrality indexes	Indexes that rank the set of nodes in a network, according to their relative importance in the specified context.	In an interacting network of i.e. pollinating insects and plant species, the insect species with highest centrality is the one "most active" in the pollination of several plant species. Conversely, a high centrality of a plant species indicates its importance to feed several insect species.	The relative importance of each node (protein or process) is obtained from centrality indexes, which rank each node in the complete network and allow the comparison between processes ("which process is more affected") by the set of represented proteins.
