# Supplementary material for: Use of Graph Theory to Characterize Human and Arthropod Vector Cell Protein Response to Infection With Anaplasma phagocytophilum
Source: Front Cell Infect Microbiol. 2018 Aug 3;8:265. doi: 10.3389/fcimb.2018.00265 (PMC6086010; doi:10.3389/fcimb.2018.00265)
Supplement: Supplementary file 2 [file Table_2.PDF]

Supplementary Table 2. Biological processes in the metabolism cluster that increased in centrality in response to infection.

Biological process (BP)	Fold change in centrality[a]	Key proteins[b]	Change in protein levels[c]
Defense response to bacteria	+53,000	B7QHS7	Over-represented
		L7M7N3	Over-represented
		Q09JR4	Infected only
Evasion or tolerance of host defense response	+2	A0A023GMN5	Infected only
		A0A023GP04	Infected only
		O77421	Uninfected only
Hippo signaling	+1,990	V5HY68	Over-represented
		V5IHG3	Infected only
Glucose catabolic process	+14,145	A0A023FXS2	Over-represented
		B7Q0R0	Over-represented
Cellular glucose homeostasis	-1,167	G3MJM6	Uninfected only
		A0A023FZH9	Uninfected only
Regulation of apoptotic process	+2	B7PB18	Over-represented
		L7LV65	Over-represented
		B7QGH7	Infected only
		V5HLW0	Infected only

[a]Fold change in centrality is shown for increase (+) or decrease (-) in infected cells when compared to uninfected controls.

[b]Key proteins refer to proteins which changes drive the increase in the centrality of these BPs.

[c]Change in protein levels refer to proteins only represented in infected or uninfected cells or over-represented in *A. phagocytophilum*-infected cells when compared to uninfected controls.
