# Supplementary material for: Use of Graph Theory to Characterize Human and Arthropod Vector Cell Protein Response to Infection With Anaplasma phagocytophilum
Source: Front Cell Infect Microbiol. 2018 Aug 3;8:265. doi: 10.3389/fcimb.2018.00265 (PMC6086010; doi:10.3389/fcimb.2018.00265)
Supplement: Supplementary file 8 [file Image_3.PDF]

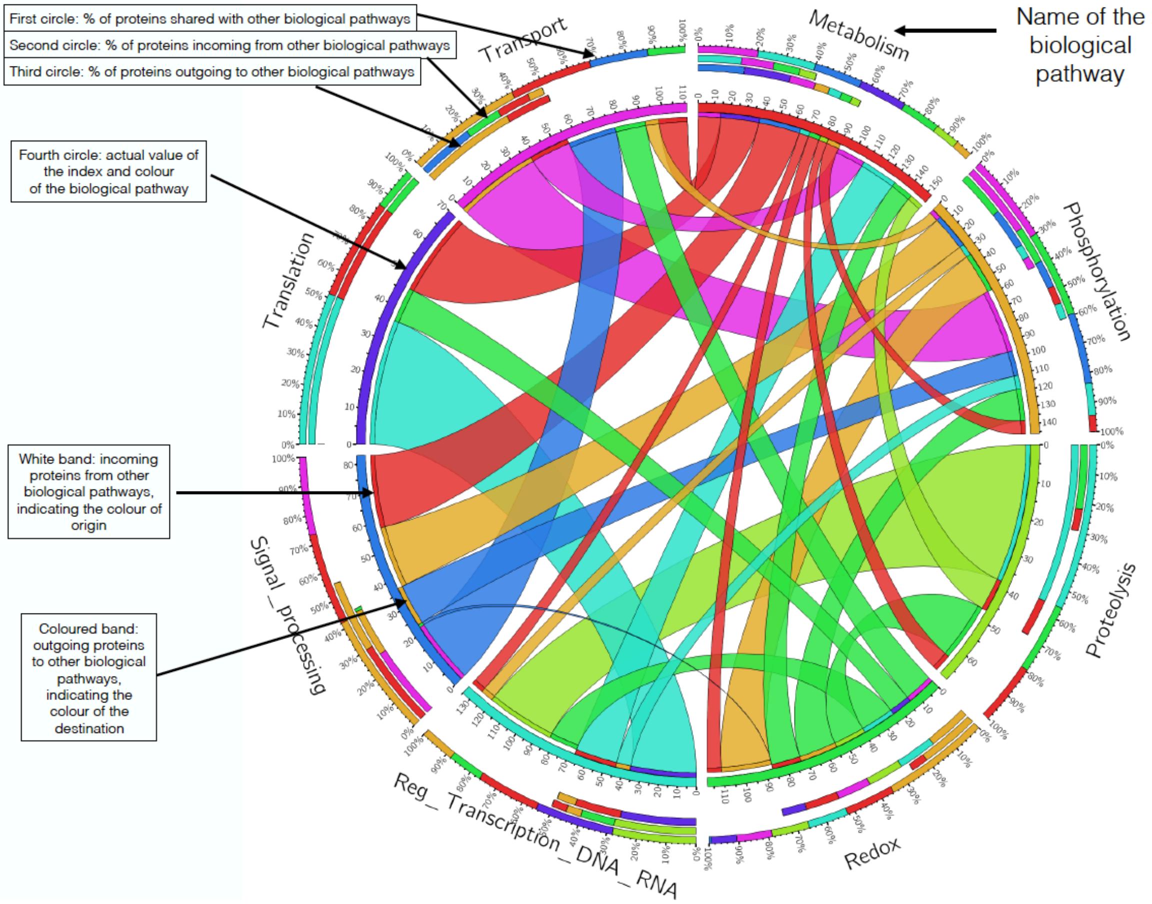

Supplementary Figure 3. Interpreting the graphical representation of the proteins shared between different biological pathways in UtC and ItC. The circle layout is a translation of a two-entry table and the values at the intersection of each row-column, representing two pathways. Colors are random and used only to identify the row and column. The ribbons show the percent of proteins ascribed to a biological pathway, and that are also involved in other pathways.
