# Supplementary material for: Use of Graph Theory to Characterize Human and Arthropod Vector Cell Protein Response to Infection With Anaplasma phagocytophilum
Source: Front Cell Infect Microbiol. 2018 Aug 3;8:265. doi: 10.3389/fcimb.2018.00265 (PMC6086010; doi:10.3389/fcimb.2018.00265)
Supplement: Supplementary file 9 [file Image_4.PDF]

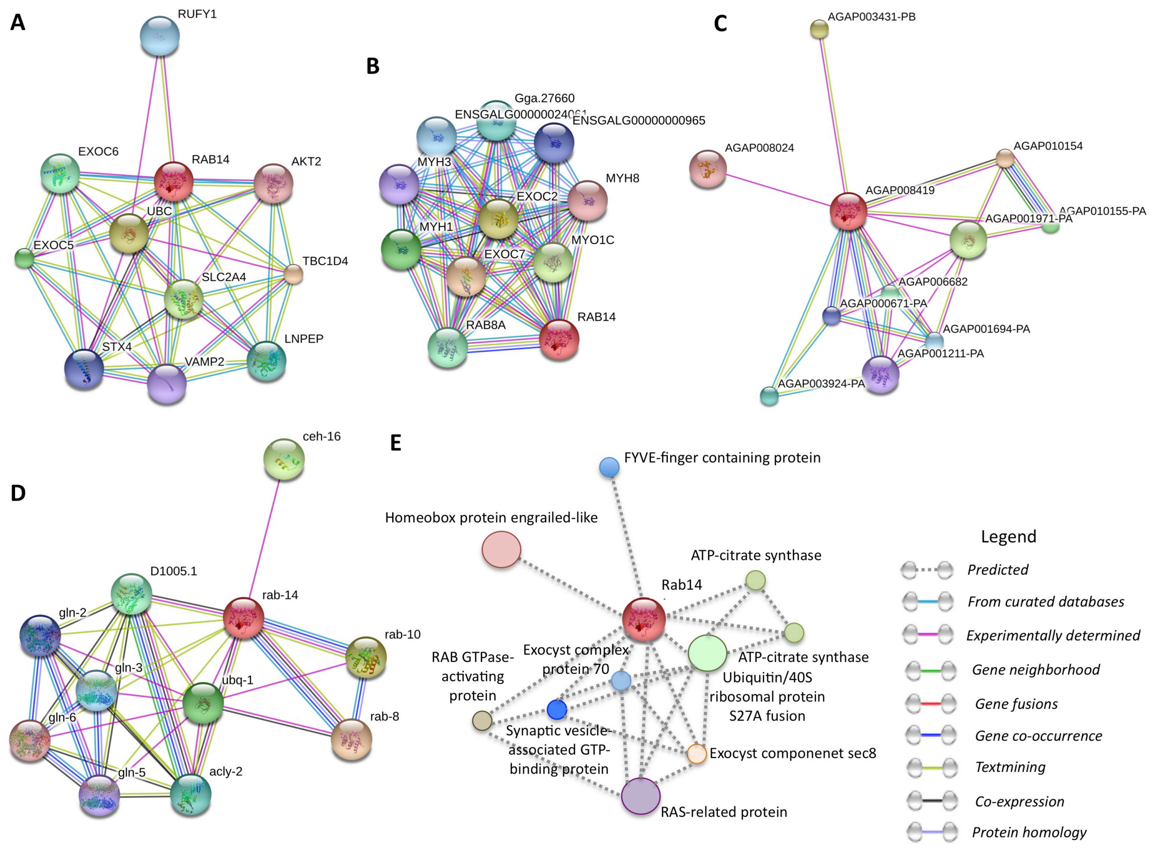

Supplementary Figure 4. The *I. scapularis* ras-related protein Rab14 interactome. The proteins interacting with the ras-related protein Rab14 (red node) in the model organisms (A) *Homo sapiens*, (B) *Gallus gallus*, (C) *Anopheles gambiae*, and (D) *Caenorhabditis elegans* were compiled and visualized in a network according to STRING (http://stringdb.org). Edges represent protein-protein associations that jointly contribute to a shared function. Line shape indicates the predicted mode of action. (E) Tick homologues were obtained with BLASTP NCBI (https://blast.ncbi.nlm.nih.gov) searches and protein-protein interactions predicted based on the mosquito ras-related protein Rab14 interactome. Tick proteins correspond to putative ras-related protein rab-14 (L7M7N3), FYVE-finger containing protein (B7QFF1), Homeobox protein engrailed like (B7QFC3), Ubiquitin/40S ribosomal protein S27A fusion (B7Q5Q7), ATP-citrate synthase (V5I49, B7Q3D3), RAB GTPase-activating protein (B7PN30), RAS-related protein (B7QDB4), Synaptic vesicle-associated GTP-binding protein (B7PEJ6), Exocyst complex protein 70 (B7P4G4), and Exocyst componenet sec8 (B7P145).
