# Supplementary material for: Use of Graph Theory to Characterize Human and Arthropod Vector Cell Protein Response to Infection With Anaplasma phagocytophilum
Source: Front Cell Infect Microbiol. 2018 Aug 3;8:265. doi: 10.3389/fcimb.2018.00265 (PMC6086010; doi:10.3389/fcimb.2018.00265)
Supplement: Supplementary file 6 [file Image_1.PDF]

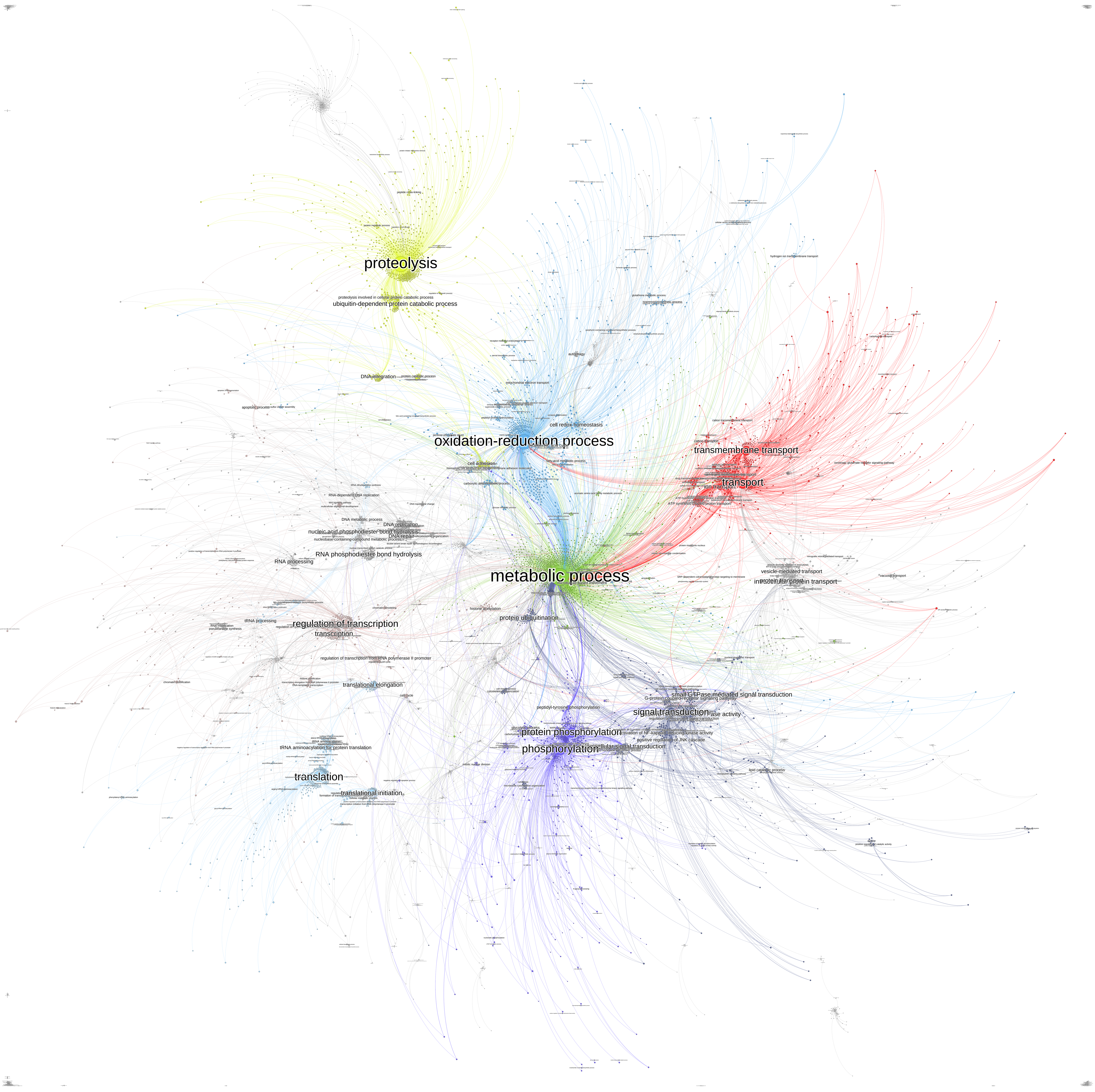

Supplementary Figure 1. The network of proteins and processes in UtC.

Only processes have been labeled to improve readability. The width of the links in proteins and processes is proportional to the representation of the protein. Colors denote clusters, or groups of nodes that interact more frequently between them than with others. The size of each node is proportional to its BNC.
